# Foot Posture Index Does Not Correlate with Dynamic Foot Assessment Performed via Baropodometric Examination: A Cross-Sectional Study

**DOI:** 10.3390/healthcare12080814

**Published:** 2024-04-10

**Authors:** Daniele Origo, Francesca Buffone, Gabriele Montini, Daniele Belluto, Marco Tramontano, Fulvio Dal Farra

**Affiliations:** 1 Department of Research, SOMA Osteopathic Institute Milan, 20126 Milan, Italy; danieleorigo@soma-osteopatia.it (D.O.); francescabuffone.ost@gmail.com (F.B.); gabriele.montini@hotmail.it (G.M.); db.studio.contact@gmail.com (D.B.); fulvio.dalfarra@unibs.it (F.D.F.); 2 Division of Pediatric, Manima Non-Profit Organization Social Assistance and Healthcare, 20125 Milan, Italy; 3 Principles and Practice of Clinical Research (PPCR), Harvard T.H. Chan School of Public Health–ECPE, Boston, MA 02115, USA; 4 Department of Biomedical and Neuromotor Sciences (DIBINEM), Alma Mater University of Bologna, 40126 Bologna, Italy; 5 Unit of Occupational Medicine, IRCCS Azienda Ospedaliero-Universitaria di Bologna, 40138 Bologna, Italy; 6 Department Information Engineering, University of Brescia, Via Branze 38, 25123 Brescia, Italy

**Keywords:** flat feet, foot morphology, gait analysis, plantar foot pressure, foot posture index, hyperpronation, pes planus

## Abstract

Background. Clinicians employ foot morphology assessment to evaluate the functionality of the method and anticipate possible injuries. This study aims to correlate static foot posture and the dynamic barefoot evaluation in a sample of healthy adult participants. Methods. The foot posture was evaluated using the Foot Posture Index-6 (FPI-6) and the dynamics were evaluated through baropodometric examination. Two operators independently assessed the participants’ foot posture through FPI-6, and then a dynamic evaluation was performed by asking them to walk 8 times across a platform. One hundred participants (mean age: 32.15 ± 7.49) were enrolled. Results. The inter-rater agreement between the two assessors was found to be excellent. The majority of the feet belonged to the 0 < FPI < 4 class (32%), followed by the 4 < FPI < 8 (31%) and the FPI > 8 ranges (19.5%). Our “area of contact” analysis showed a significant poor correlation between FPI and total foot, midfoot, and the second metatarsophalangeal joint (MTPJ) (−0.3 < r < 0). Regarding “force” parameters, the analysis showed a poor correlation between the midfoot, hallux, and the second toe (−0.2 < r < 2); finally the “pressure” analysis showed a poor correlation between FPI, the fourth MTPJ, and the second toe (−0.2 < rs < 0.3) and a moderate correlation between the hallux (r = 0.374) and the fifth MTPJ (r = 0.427). Conclusions. This study emphasizes the constrained correlation between static foot posture observation and dynamic barefoot examination.

## 1. Introduction

Clinicians utilize foot morphology assessment to gauge its functionality and anticipate potential injuries or discomfort [1]. There is a prevalent belief that deviations in static foot posture may predispose individuals to lower limb injuries, with flat foot (low medial longitudinal arch) and cavus foot (high medial longitudinal arch) being common examples of these [2]. While radiographic evaluation is the gold standard among diagnostic techniques in terms of assessing static foot posture, its limitations, such as irradiation exposure and high costs, often lead clinicians to prefer clinical measurements due to their greater practicality [3]. To address this need, Redmond and colleagues [4] developed and validated the Foot Posture Index 6 items (FPI-6), a six-item scoring tool that assesses rearfoot and forefoot through palpation [5]. The FPI-6 has been widely utilized with both adults [2] and children [6], is considered a reliable method for measuring foot posture, and is extensively used in clinics and research [7]. Moreover, it has been cross-culturally adapted and validated in various versions, including Italian [8]. On a different note, baropodometric examination has shown good reliability in assessing both static and dynamic foot functions during gait assessments [9,10]. However, evidence regarding the association between static foot morphology, foot dynamics, and musculoskeletal injuries remains scarce [11]. Despite being easily applicable, some studies have highlighted the poor inter-rater reliability of the FPI and its limitations in predicting dynamic foot function [12,13]. Redmond and colleagues [4] found that the FPI predicted only 41% of ankle frontal plane motion during midstance, while Nielsen and colleagues [14] reported that FPI predicted 45% of minimal navicular height and 13.2% of navicular drop during walking, indicating limitations in terms of predicting midfoot dynamics on a case-by-case basis. Conversely, Chuter and colleagues [15] found the FPI to have a strong precitive capacity (85%) in assessing maximum rearfoot eversion. A recent meta-analysis [7] also indicated a weak relationship between pronated foot posture, medial tibial stress syndrome, and patellofemoral pain, suggesting that static measures may have limitations in terms of predicting foot kinematics. Other factors such as BMI, female gender, excessive hip external rotation, quadriceps weakness, and patellar instability seem to be more implicated in these painful conditions [16,17]. Given the clinical significance of the FPI and other static foot assessments [7,11,18], and considering the aforementioned issues, we hypothesize that FPI assessment may not accurately reflect the dynamic function of the foot in terms of contact area, force, and peak plantar pressure. Therefore, the primary aim of this study is to determine the correlation between static foot posture, measured with the FPI, and dynamic barefoot evaluation, assessed through baropodometry, in a sample of healthy adult participants. The secondary aim is to assess the inter-rater agreement of the FPI-6 to enable its use among the operators involved in the assessment procedures of this study.

## 2. Materials and Methods

### 2.1. Study Design and Setting

The results of this cross-sectional study were reported according to the “Strengthening the reporting of observational studies in epidemiology” (STROBE) statement [19]. Foot posture was evaluated using the FPI and via baropodometric examination, with information obtained concerning foot contact areas, forces, and pressures. This study was carried out at SOMA Institute, a tertiary outpatient clinical center in Milan (Italy), from May 2021 to May 2022. 

### 2.2. Ethical Approval

This study was approved by the institutional review board of SOMA Institute and given protocol number SIOM-AA0017. Prior to participation, all participants were provided with detailed information about the research, including its voluntary nature, and were assured of the anonymity of their data when featured in reports and publications. All procedures conducted in this study adhered to the ethical standards outlined in the relevant national and institutional guidelines on human experimentation, as well as the principles laid out in the World Medical Association Declaration of Helsinki. All participants provided written informed consent.

### 2.3. Participants

Following the lead of similar studies that involved no more than 85 participants [20,21], we recruited a convenience sample of 100 healthy adults from among the students and personnel of the clinic after announcing the study. Those interested in participating voluntarily contacted the outpatient office, where eligibility was confirmed by a physiotherapist who was unaware of our research objectives. Inclusion criteria included individuals aged between 20 and 60 years of both genders and without musculoskeletal pain (VAS score of less than 3). Exclusion criteria included the presence of post-traumatic, neurological, genetic, rheumatological, and neoplastic pathologies. 

### 2.4. Study Protocol

All the participants attended a single daily session to collect data concerning static foot posture and dynamic function. The participants underwent steps prior to the static assessment and subsequent to the functional test on the baropodometric platform. 

To assess foot posture, a podiatrist and a physiotherapist, both with over 10 years of experience and affiliated with the unit of the clinic specializing in musculoskeletal disorders, independently evaluated the participants using the FPI-6. Before data collection, the operators underwent consensus training. This consisted of three sessions, each lasting two hours. In the first two meetings, the operators familiarized themselves with the rating procedure, and in the final session they addressed any potential concerns. During the assessment, participants were instructed to stand quietly on both feet and keep their arms relaxed along their bodies, close to their thighs, and their heads in a neutral position, in accordance with international standardization criteria for baropodometric tests [22]. Additionally, walking trials were conducted in a laboratory setting along a 10 m pathway. Participants were instructed to walk barefoot, albeit comfortably, along the walkway at their normal speed [23]. The walk began 2 m before the walkway to account for gait initiation issues and concluded 2 m after the walkway to adjust for changes in speed at the end. Each participant repeated the trial four times in each direction [24].

When selecting the walking performance to record, the assessors were instructed to choose instances where the entire foot was completely in contact with the platform. If there were multiple proper footprints, one was randomly selected for analysis. In the event of disagreement between the assessors, they were required to consult a third evaluator, a physiotherapist with more than 10 years of experience, to resolve the discrepancy. The Emed^®^ n50 platform https://www.novelusa.com/emed (accessed on 10 April 2021) was used to assess foot function. This medical device uses calibrated capacitive sensors (2 sensors/cm^2^), allowing it to record area, force, and pressure distributions in both static and dynamic conditions. The data were analyzed using Emed Professional Software https://www.novelusa.com/emed (accessed on 10 April 2021). 

### 2.5. Statistical Analysis

Descriptive statistics were used to report the general characteristics of the sample and we obtained measurements via baropodometric examination (mean and standard deviation; mode, median, and quartiles for continuous variables; and frequencies for categorical variables). The normality of the FPI-6 class distribution was verified using the Shapiro–Wilk test. The ICC, using a two-way mixed-effects model for absolute agreement within its 95% confidence interval (CI), was considered in order to assess the reliability. ICCs were interpreted as follows: 0.81–1.00 as perfect, 0.61–0.80 as substantial, 0.41–0.60 as moderate, 0.21–0.40 as fair, and 0.00–0.20 as light. 

The correlation between the FPI-6 and baropodometric examination was assessed using the Spearman correlation test. The interpretation of the correlation coefficient (r) is as follows: when r is close to +1, it indicates a positive correlation; when it is close to −1, it indicates a negative correlation; and when it is close to 0, it suggests the absence of a linear relationship. Specifically, the interpretation of the “r” coefficient for both positive and negative values is as follows: 0.1–0.19 indicates no or negligible correlation; 0.20–0.29 suggests a weak correlation; 0.30–0.39 indicates a moderate correlation; 0.40–0.69 suggests a strong correlation; and r ≥ 0.70 indicates a very strong relationship [25]. The choice of the parameters under consideration in the correlation test was based on Buildt’s work [2], and we specifically considered the variables used in the baropodometric analysis that appeared to show a linear correlation with FPI-6.

A *p*-value ≤ 0.05 was considered to show statistical significance. All calculations were performed using SPSS software version 24.0.

## 3. Results

One hundred participants were recruited with a mean age of 32.15 ± 7.49 years, resulting in a total of 200 feet being analyzed. The average BMI of the participants was 22.45 ± 2.42 kg/m^2^. The demographic characteristics of the sample are reported in Table 1.

The inter-rater agreement between the two operators was excellent, as the ICC was 0.97 (0.948; 0.983). The distribution of the examined feet across the different classes of the FPI scores is reported in Table 1. The majority of the feet belonged to the 0 < FPI < 4 class (32%), followed by the 4 < FPI < 8 (31%) and by the FPI > 8 ranges (19.5%). 

All the data collected from the dynamic barefoot assessment are reported in Table 2, Table 3 and Table 4. 

As regards the “area of contact”, three out of the four analyses of correlation resulted in statistical significance (*p* ≤ 0.05), showing a poor correlation between FPI and total foot, the midfoot, and the second metatarsophalangeal joints (MTPJ) (−0.3 < r < 0). The analyses of the correlations between FPI and the “force” parameters considered them to be statistically significant (*p* ≤ 0.05). In greater detail, a poor correlation was detected when considering midfoot, hallux, and the second toe (−0.2 < r < 2). A moderate correlation was found with the fifth MTPJ (r = −0.477). Concerning pressure, the analysis brought significant results with four out of the five parameters considered. In more detail, there was poor correlation between the FPI, the fourth MTPJ, and the second toe (−0.2 < r < 0.3). A moderate correlation was present in the cases of the hallux (r = 0.374) and the fifth MTPJ (r = 0.427). The results of the correlation analysis are reported in Table 5. 

## 4. Discussion

The primary aim of this study was to identify the correlation between static foot posture, measured by using the FPI-6, and dynamic barefoot evaluation in healthy adult participants. Secondarily, we tested the inter-rater reliability of FPI-6 to provide proof of its use among the operators involved in this study, following consensus training methods. According to the metrics of previous studies [5,26], the majority of the observed feet were classified as “normal”, followed by “pronated” and “very pronated”, with only a small portion categorized as “supinated” or “very supinated”. These results were supported by excellent inter-rater reliability, suggesting the potential benefits of consensus training in terms of enhancing FPI reliability [8]. Our main findings indicate that the FPI should not be regarded as an indicator of dynamic foot function. Specifically, the expected correlation between the FPI and baropodometric examination was consistently weak for parameters related to foot contact area, force, and pressure, with occasional moderate correlation values observed. Therefore, foot posture observation cannot reliably determine foot function during walking. These results confirm the hypothesis upon which this study was based. In view of the extensive data collected, this discussion focuses on pertinent findings with clinical implications. Firstly, our results provide evidence of potential distinctions between a pronated foot and a flat foot, despite the possibility of their coexistence [27]. Regarding the midfoot-related results in our dataset, parameters such as contact area, forces, and peak plantar pressure exhibited the lowest values in the maximum pronation score group (FPI > 8), contrasting with the higher values reported in flat feet by Buildt and colleagues [2]. Moreover, while Buildt’s study indicated a linear increase in values from lower to higher FPI scores, our study revealed the opposite trends for pressures and forces and irregular trends for contact area values.

Another discrepancy arose in the parameter “total area of the foot”. In a scenario where pronated and flat feet were equivalent, the foot surface area would be largest in both static and dynamic conditions, following a linear increasing tendency from a cavus condition (FPI < −4) to a flat condition (FPI > 8). However, during our dynamic test, the foot’s total area did not exhibit a linear trend, resulting in a poor correlation index, with the highest value observed in the normal foot FPI class (0 < FPI < 4).

Finally, our findings partially diverged from those of other authors concerning the first metatarsophalangeal joint (1st MTPJ) and the hallux, with varying values and trends noted in the correlation analysis across different FPI classes. However, the imprecision of the estimates (high standard deviations) prevents us from considering these data to be robust. One of the possible explanations such discrepancies could be referrable to the different foot posture assessments implemented in the various studies: to give some examples, Buildt and colleagues [2] considered the “arch index” and the “normalised navicular height truncated” in addition to FPI-6. Other authors adopted footprint analysis [28]. However, the use of FPI-6 should not be used to define classes such as “planus” or “cavus”, since it is based on the pronation or supination degree; as stated above, these conditions do not always coincide [19].

These findings are proof of the limitations of FPI use in predicting foot dynamics in adults, requiring a reconsideration of this tool’s clinical use. While diagnostic examinations guide clinical decisions, they must possess good reliability and validity values [4]. Reliability studies on FPI-6 [29,30] have shown conflicting results, with concerns raised about the index’s cumulative nature, leading to general ambiguity [19]. Inter-rater reliability was poor across all investigated groups (children, adolescents, and adults), consistent with the findings obtained by other authors [31,32,33,34]. Concurrent validity studies by Redmond et al. [4], comparing FPI-6 with electromagnetic motion tracking and 2D-video sequence analysis [11], revealed limited predictive capabilities for foot dynamics, particularly for midfoot movement during contact [9]. Our findings are in line with previous studies [9,11] indicating a weak correlation with dynamic assessment. They are contrary to the results in Teyhen et al.’s study [26], which demonstrated a positive association with plantar pressure measurements. 

These observations highlighted the incomplete validity of FPI-6 in predicting foot function, given the weak association between static foot type and dynamics and the complex relationships between foot joints [35,36]. Considering the foot’s intrinsic dynamic nature, static assessments often fail to fully capture its dynamic morphology during motion [37,38], which is explained by the FPI’s inability to distinguish rigid from flexible flat feet [19].

Although some studies use FPI as an assessment tool for flat foot or high-arched feet [2,3,39], it is crucial to recognize that foot pronation, rearfoot eversion, and flat foot are not synonymous [40]. The complex relationship between foot pronation, flat foot, and cavus foot requires further investigation, with consideration of biomechanical, genetic, and environmental factors [41,42,43,44]. In summary, the FPI-6 is a valuable tool for static postural assessment of the foot, providing a score on the basis of the pronation–supination degree. However, it cannot predict dynamic gait strategies, such as areas of loading and overloading. It could be important to supplement it with a dynamic gait assessment and performance tests (e.g., single-leg squat or single-leg balance) [45]. Additionally, a comprehensive clinical examination of the spine, pelvis, and lower limbs might be relevant [46].

Accurate foot shape and function definitions influence decision making and therapeutic options, impacting healthcare systems, professionals, and patients [47,48,49]. Incorporating diverse assessments, like dynamic gait analysis or biomechanical assessments, can enhance our understanding of foot function and improve treatment outcomes. Furthermore, educating patients about the limitations of static foot posture assessment is essential, highlighting the multifaceted nature of foot evaluation for both physical and psychological reasons. Lastly, researchers should perfect detailed clinimetric properties of FPI, especially in terms of validity [50], while future research should prioritize the dynamic assessment of foot function. Our study displays some limitations that should be taken into consideration in order to better interpret the results.

Firstly, we did not perform a sample size calculation, which means that we cannot be entirely confident about the representativeness of the actual population. However, as mentioned previously, our sample exhibited foot characteristics comparable to those of the general population in terms of posture distribution.

Secondly, although we defined specific selection criteria for our sample, we did not differentiate between various foot clinical conditions, which could have influenced the observed correlations. Future research focusing on specific foot conditions could offer additional insights.

Lastly, as an inherent limitation of the examination, a single dynamic assessment might not fully capture the variability of foot function across different activities and conditions. Moreover, force and pressure during gait are influenced by spatio-temporal parameters such as velocity, cadence, and step length. Conducting multiple sessions could provide a more comprehensive understanding of the relationship between static foot posture and dynamic function. 

One significant aspect of this study is its demonstration that static foot posture and dynamic foot function can vary significantly, potentially impacting clinical decision making. Furthermore, our findings suggest avenues for further research exploring the intricate relationship between bodily structure and function.

## 5. Conclusions

This study emphasizes the constrained correlation between static foot posture observation and dynamic barefoot examination. The FPI-6 showed weak validity in terms of predicting dynamic foot function. Additional research is warranted to explore how different foot conditions and pathologies may impact the relationship between static posture and dynamic function.

## Figures and Tables

**Table 1:** Demographic characteristics of the participants and inter-rater agreement between the two operators.

N. of Participants	100
Female/male	39/61
Weight (kg)	68.3 ± 13.1
Height (cm)	173.7 ± 9.08
BMI (kg/m^2^)	22.45 ± 2.92
Mean age	32.15 ± 7.49
FPI > 8 (%)	39 (19.5)
4 < FPI < 8 (%)	62 (31)
0 < FPI < 4 (%)	64 (32)
−4 < FPI < 0 (%)	25 (12.5)
FPI < −4 (%)	10 (5)
ICC (95% CI)	0.97 (0.948; 0.983)

BMI: body mass index; FPI: foot posture index.

**Table 2:** Area of contact (cm^2^) results during dynamic assessment.

FPI Range	Total	Heel	Midfoot	1st MTPJ	2nd MTPJ	3rd MTPJ	4th MTPJ	5th MTPJ	Toe 1	Toe 2	Toe 3,4,5
FPI > 8	137.54 (20.59)	35.54 (4.52)	24.27 (9.74)	12.92 (2.28)	10.38 (1.73)	11.6 (1.71)	10.16 (1.38)	6.80 (0.97)	13.17 (1.96)	5.1 (1.03)	7.44 (2.75)
4 < FPI < 8	140.82 (19.67)	36.40 (4.23)	25.11 (8.72)	13.38 (2.26)	10.64 (1.70)	12.06 (1.53)	10.41 (1.31)	7.01 (0.95)	12.91 (2.15)	5.25 (1.48)	7.50 (3.25)
0 < FPI < 4	144.70 (19.34)	37.09 (4.66)	26.94 (7.73)	13.59 (2.20)	10.96 (1.59)	12.15 (1.67)	10.74 (1.47)	7.27 (1.01)	13.09 (2.22)	5.19 (1.21)	7.54 (2.84)
−4 < FPI < 0	152.97 (16.45)	39.12 (4.28)	28.47 (7.11)	15.10 (2.21)	11.46 (1.25)	13.17 (1.78)	11.34 (1.55)	7.90 (1.00)	13.50 (1.93)	5.22 (1.30)	7.65 (3.68)
FPI < −4	142.59 (10.84)	36.98 (4.98)	24.97 (5.27)	13.83 (1.91)	10.85 (1.14)	12.33 (1.18)	10.65 (1.38)	7.38 (0.89)	14.29 (1.26)	5.03 (1.07)	6.23 (2.61)

MTPJ: metatarsophalangeal joint; FPI: foot posture index.

**Table 3:** Force (N) results during dynamic assessment.

FPI Range	Total	Heel	Midfoot	1st MTPJ	2nd MTPJ	3rd MTPJ	4th MTPJ	5th MTPJ	Toe 1	Toe 2	Toe 3,4,5
FPI > 8	672.19 (139.8)	437.82 (89.5)	82.58 (51.41)	114.28 (55.57)	148.93 (44.74)	165.81 (36.5)	94.48 (24)	38.12 (14.9)	133.92 (48.84)	23.52 (10.1)	22.86 (13.5)
4 < FPI < 8	663.31 (134)	439.63 (88.7)	92.34 (50.78)	119.68 (68.86)	154.62 (55.81)	170.62 (48.42)	103.98 (36.5)	48.08 (25.03)	124.48 (84.97)	25.89 (16.6)	24.59 (17.6)
0 < FPI < 4	710.07 (148.5)	455.14 (96.3)	114.47 (58.07)	117.54 (47.77)	171.49 (55.62)	167.71 (45.52)	112.97 (37.8)	59.8 (26.85)	103.59 (41.28)	23.16 (17.7)	21.67 (17.5)
−4 < FPI < 0	746.08 (130.65)	475.48 (79.7)	127.07 (66.82)	145.94 (58.47)	160.71 (43.34)	171.63 (41.27)	118.7 (27.7)	67.5 (23.75)	89.76 (27.41)	21.29 (12.1)	23.37 (18.3)
FPI < −4	730.55 (133.22)	466.78 (91.73)	118.18 (67.73)	127.45 (62.08)	152.12 (52.6)	174.24 (42.18)	127.03 (31.69)	79.99 (19.33)	99.68 (27.61)	18.98 (9.19)	21.14 (11.6)

MTPJ: metatarsophalangeal joint; FPI: foot posture index.

**Table 4:** Pressure (KPa) results during dynamic assessment.

FPI Range	Total	Heel	Midfoot	1st MTPJ	2nd MTPJ	3rd MTPJ	4th MTPJ	5th MTPJ	Toe 1	Toe 2	Toe 3,4,5
FPI > 8	556.43 (204.73)	342.53 (85.3)	94.82 (36.4)	224.34 (138.9)	373.56 (119.7)	384.74 (158.4)	232.16 (57.29)	141.99 (72.7)	444.84 (198.8)	109.4 (40.9)	69.9 (31.5)
4 < FPI < 8	487.83 (141.3)	336.25 (94.72)	99.57 (33.5)	198.54 (85.47)	357.88 (98.34)	359.72 (109.8)	247.03 (87.63)	183.88 (149.2)	339.28 (149.7)	113.4 (55.7)	71.9 (42.3)
0 < FPI < 4	501.19 (131)	324.03 (86.21)	108.46 (34.7)	211.77 (83.62)	388.41 (146.9)	352.18 (95.23)	262.44 (84.43)	239.21 (141.8)	290.07 (143.4)	99.03 (41.7)	64.29 (30)
−4 < FPI < 0	460.51 (108.8)	311.48 (53.53)	117.69 (53.4)	244.86 (133.8)	336.02 (103.1)	346.02 (107.5)	262.11 (53.52)	250.12 (130.9)	247.94 (97.84)	93.84 (49.9)	64.55 (34.9)
FPI < −4	532.57 (123.9)	338.48 (67.36)	123.63 (77.9)	251.06 (156.9)	317.12 (89.11)	360.91 (101.9)	266.06 (48.85)	357.27 (129.1)	246.51 (101.9)	76.96 (34.6)	57.27 (35.6)

MTPJ: metatarsophalangeal joint; FPI: foot posture index.

**Table 5:** Results of the correlation analysis (coefficient “r”) between FPI-6 score and area (cm^2^), force (N), and pressure (kPa) parameters.

	Area	Force	Pressure
Parameter			
Total Area	−0.218 *		
Midfoot	−0.141 *	−0.254 **	
1st MTPJ			−0.93
2nd MTPJ	−0.225 *		
4th MTPJ			−0.179 *
5th MTPJ		−0.477 **	−0.427 **
Toe 1		0.283 **	0.374 **
Toe 2	0.025	0.142 *	0.209 *

*: *p* < 0.05; **: *p* < 0.001; MTPJ: metatarsophalangeal joint.

## Data Availability

Data are available upon reasonable request to the corresponding author.
